# Identification of B-Cell Epitopes Located on the Surface of the S1 Protein of Infectious Bronchitis Virus M41 Strains

**DOI:** 10.3390/v17040464

**Published:** 2025-03-24

**Authors:** Zichen Gao, Jianing Hu, Yiqin Cai, Ye Liu, Guihu Yin, Xinyu Guo, Ruiying Wang, Meng Zhong, Qingtao Liu, Xiuli Feng

**Affiliations:** 1 Key Laboratory of Animal Microbiology of China’s Ministry of Agriculture, College of Veterinary Medicine, Nanjing Agricultural University, Nanjing 210095, China; 2022107058@njau.edu.cn (Z.G.); 2021207047@stu.njau.edu.cn (J.H.); 2021107050@stu.njau.edu.cn (Y.C.); 2023007051@stu.njau.edu.cn (Y.L.); yinguihu@stu.njau.edu.cn (G.Y.); 2022807117@stu.njau.edu.cn (X.G.); 2023107109@stu.njau.edu.cn (R.W.); 2023807130@stu.njau.edu.cn (M.Z.); 2 MOE Joint International Research Laboratory of Animal Health and Food Safety, College of Veterinary Medicine, Nanjing Agricultural University, Nanjing 210095, China; 3 Key Laboratory of Veterinary Biological Engineering and Technology, Ministry of Agriculture, Institute of Veterinary Medicine, Jiangsu Academy of Agricultural Sciences, Nanjing 210014, China

**Keywords:** avian infectious bronchitis virus, monoclonal antibody, S1 antigenic determinants, B-cell epitope, peptide scanning, overlapping fragments, prokaryotic expression

## Abstract

Avian infectious bronchitis is caused by the avian infectious bronchitis virus (IBV), which poses a significant threat to the poultry industry and public health. The S1 protein of IBV plays a crucial role in the process of the virus invading host cells. To investigate the significant antigenic targets within the S1 protein, in this study, the truncated S1 sequence of the IBV M41 strain was cloned with approximately 660 bp and expressed. After purification and renaturation, the recombinant S1 protein was immunized into BALB/c mice. Then, following fusion with lymphocytes and SP2/0 cells, the indirect ELISA and Western blotting techniques were employed to screen hybridoma cell lines secreting monoclonal antibodies (mAbs) targeting the S1 protein. Antigenic epitopes of the mAbs were identified using truncated S1 fragments and peptide scanning. The results indicated that three hybridoma cell lines stably secreting S1 protein-specific mAbs (2A10, 4E9, and 5E12) were screened. The heavy chains of the three mAbs were IgG1, and all three mAbs contained kappa light chains. The identified minimal B-cell epitopes were ^132^RVSAMK^137^ and ^142^FYNLTV^147^. Homology analysis showed these both epitopes were conserved across IBV subtypes and located on the S1 protein surface. The conserved β-sheet epitope ^132^RVSAMK^137^ and the surface-exposed, flexible loop epitope ^142^FYNLTV^147^ serve as ideal targets for broad-spectrum diagnostics and early infection detection, respectively. These epitopes provide unique structural advantages for antibody binding, enabling the design of multivalent epitope vaccines or the development of immunomodulatory drugs. They offer novel biomaterials and targets for antibody-based drug development and rapid detection methods for avian infectious bronchitis virus (IBV), holding significant potential for the prevention and control of IBV.

## 1. Introduction

Avian infectious bronchitis (IB) is an acute and highly contagious respiratory disease caused by the avian infectious bronchitis viruses (IBV) of the genus *Coronavirus*, but over the past decade, the incidence of various viral pathogens in birds and mammals has increased [1,2,3]. IBV was first identified in the 1930s as the inaugural described coronavirus, and its viral etiology was established in 1936 [4,5]. In 1980, IBV was first reported in China, isolated from chickens, and then endemic to the respiratory system of diseased poultry. Moreover, some IBV strains may also have an impact on the reproductive and urinary systems [6]. Due to the absence of proofreading capability in IBV’s RNA polymerase, genetic mutations and recombinations are likely to occur during replication. This results in a lack of cross-protection among different serotypes of IBV, leading to recurrent outbreaks globally and imposing significant economic losses on the global poultry industry [7]. Currently, through comparative analysis of genetic differences of the sequences from various IBV isolates, IBV has been classified into 32 phylogenetic lineages (G-I 1–27, G-II, GIV-GVI) [8].

IBV is an enveloped virus with a positive-sense single-stranded RNA genome measuring 27.6 kb. The 3′ terminal region of the viral genome encodes structural proteins, including the spike protein (S), envelope protein (E), membrane protein (M), and nucleocapsid protein (N) [9]. S protein is a type I glycoprotein and serves as the primary antigenic component of coronaviruses. Upon binding to receptors on target cells, the S protein facilitates the fusion of the virus with the host cell [10]. There is ample evidence to prove that N-glycosylation of the viral glycoprotein affects the ability of the virus to infect host cells and evade recognition by the host immune system [11]. The S protein has extensive glycosylation modifications at aspartic acid residues and undergoes conformational changes, which are particularly critical during the host cell infection process [12,13]. The S protein is cleaved by a furin-like protease at the highly alkaline pentapeptide motif RRFRR into the S1 and S2 subunits [14]. This characteristic of the S protein has been demonstrated to enhance membrane fusion [15]. The variable S1 subunit serves as the principal immunogen and plays vital roles, including the binding of host cell receptors, the generation of neutralizing antibodies and inhibitory hemagglutinin antibody epitopes, and host tropism [16]. The conserved S2 subunit mediates the fusion of the viral particle with the cell membrane. The S1 protein comprises two principal structural domains, namely the N-terminal domain (NTD) and the C-terminal domain (CTD) [17]. The S1-NTD of IBV contains a receptor-binding domain (RBD), which interacts with the sugar receptors on the surface of host cells [18]. Research has demonstrated that the interaction between the S1-NTD of the IBV M41 strain and α-2,3-linked sialic acid on the surface of primary chicken embryonic kidney cells is a critical determinant for IBV entry and infection [19]. Moreover, N-glycosylation at residues 51, 77, 103, 144, 178, and 212 within the S1-NTD is essential for its interaction with chicken tracheal epithelial cells [20]. The IBV S1-NTD possesses distinctive structural characteristics compared to those of other coronaviruses and plays an important role in the initial receptor engagement and conformational changes during the virus entry process, which might affect receptor utilization and membrane fusion functions [21]. IBV S1-CTD primarily mediates the binding to protein receptors [22]. This suggests that the S1 protein may be of great significance during coronavirus infection, particularly in the process of recognition and binding to the host cell receptor. However, the underlying functional mechanism and key target of S protein in IBV on specific receptor recognition remain to be elucidated.

Since the discovery of IBV, its genetic diversity has resulted in the continuous emergence of new genotypes and serotypes [23]. Meanwhile, the S protein in IBV possesses the features of antigenicity and variability and serves as the primary target for the generation of neutralizing antibodies. Hence, the specific epitopes can be employed as vital immunogens and antiviral targets. The S1-CTD RBD region has been extensively studied as the primary target for monoclonal antibodies. Despite the critical role of the S1-NTD in the early stages of viral infection, its epitopes and functional mechanisms remain relatively underexplored. This study focuses on the preparation of monoclonal antibodies targeting the S1-NTD, aiming to identify new epitopes and elucidate their structural and functional characteristics. The truncated S1 gene was cloned and expressed in the prokaryotic system. The monoclonal antibodies against the S1 protein were screened after cell fusion with SP2/0 cells and spleen cells from the mice immunized with the recombinant S1 protein. B-cell epitopes on the S1 protein were identified by constructing overlapping truncated fragments. The secondary structure of the epitopes was clarified through homology and modeling predictions, and their functional characteristics were analyzed, revealing their unique advantages in antibody binding.

## 2. Materials and Methods

### 2.1. Virus and Cells

The IBV M41 strain used in this study was gifted by Professor Zhang Xiaorong from Yangzhou University (Yangzhou, China). It was obtained from 9-day-old SPF embryonated eggs injected with the virus, and the allantoic fluid was harvested and stored at −80 °C.

SP2/0 cells were cultured in RPMI-DMEM medium (VivaCell, Shanghai, China) with 20% fetal bovine serum (FBS, VivaCell, Shanghai, China) and 100 U/mL penicillin, and 0.1 mg/mL streptomycin at 37 °C and 5% CO_2_.

The CEK cells were isolated from 18-day-old SPF chicken embryos and cultured in M-199 medium (VivaCell, Shanghai, China) with 10% FBS, 100 U/mL penicillin, and 0.1 mg/mL streptomycin at 37 °C and 5% CO_2_.

### 2.2. S1 Gene Cloning and Recombinant Protein Expression

Based on the amino acid sequence of the S1 gene from GeneBank (AY561711.1), the antigenic and conserved regions of amino acids were analyzed using DNASTAR 11 software, which was selected for truncated expression (amino acids 38-257). Targeted primers for the truncated S1 gene were designed using SnapGene 6.0.2, as detailed in Table 1. Total viral RNA of the IBV M41 strain was extracted from the allantoic fluid using Trizol reagent (Takara Bio, Tokyo, Japan), and subsequently reverse transcribed into cDNA, which served as the template for PCR amplification using PrimeSTAR HS DNA Polymerase (Takara Bio, Tokyo, Japan), following the reactions and procedures outlined in Table 2 and Table 3. The PCR amplification was identified and purified using agarose gel electrophoresis, followed by ligation into the pCold I plasmid with restriction endonucleases *Kpn* I and *Hind* III. Following identification by PCR and double enzyme digestion, the positive recombinant plasmid pCold I-S1 was transformed into the *E. coli* BL21 (DE3) strain and induced with 1 mM isopropyl-β-d-galactopyranoside (IPTG) at 16 °C for 24 h. The induced precipitates were lysed with 8 M urea buffer (50 mM Tris, 0.05M NaCl, and 8 M urea), and the expression form of recombinant protein was analyzed using sodium dodecyl sulfate-polyacrylamide gel electrophoresis (SDS-PAGE) and Western blotting. The recombinant S1 protein was purified using nickel column affinity chromatography, followed by dialysis and concentration to yield the final purified product. The concentration of the recombinant protein was quantified using the BCA assay.

### 2.3. Immunization of Mice

Five 6–8-week-old female Balb/c mice were initially immunized with the purified recombinant S1 protein. The protein was emulsified with Freund’s Complete Adjuvant at an equal volume ratio of 1:1. Each mouse was intraperitoneally injected with a dose of 100 μg of S1 protein. The second and third immunizations were carried out with Freund’s incomplete adjuvant, with a 2-week interval between each immunization. One week after the third immunization, the serum antibody titer of the immunized mice was determined by indirect ELISA employing nickel column-purified S1 recombinant protein encapsulation. The mouse exhibiting the highest antibody level was selected for booster immunization with 200 μg of recombinant S1 protein prior to cell fusion.

### 2.4. Preparation and Identification of Monoclonal Antibodies

One day before cell fusion, the peritoneal macrophages from 8-week-old ICR mice were obtained as feeder cells. At the time of cell fusion, the splenocytes were isolated from booster mice and were fused with SP2/0 cells at a ratio of 5:1 using PEG4000 solution (SIGMA, St. Louis, MO, USA). The fused cells were resuspended in DMEM selection medium containing 20% FBS and HAT, and added into 96-well plates containing feeder cells for culturing. Ten days after fusion, 100 μL of supernatant in fusion cells was removed, and 100 μL of HT-supplemented medium was added. The antibody level in the supernatant was detected by indirect ELISA to screen for positive clones. After 3 to 4 rounds of subcloning, the stable and specific positive hybridomas were obtained. To prepare high-titer monoclonal antibodies, 8-week-old Balb/c mice were primed by intraperitoneal injection of 500 μL of ascites-specific adjuvant (Biodragon, Suzhou, China). One week later, 1 − 2 × 10^6^/mL hybridoma cells were injected intraperitoneally into each mouse to prepare the ascites fluid monoclonal antibody. The type and isotype of the monoclonal antibody were determined using a mouse monoclonal antibody isotype identification kit (Pronteintech Group, Rosemont, IL, USA). Finally, the specificity of the selected monoclonal antibody was determined by Western blotting and indirect immunofluorescence.

### 2.5. Enzyme-Linked Immunosorbent Assay (ELISA)

The recombinant protein S1 was diluted to 2 μg/mL using a carbonate buffer (0.05 mol/L, pH = 9.6), and 100 μL of the diluted solution was added to ELISA plate and incubated overnight at 4 °C. After blocking with 5% skimmed milk at 37 °C for 2 h, the test serum samples, mouse negative serum, and positive serum were added at 100 μL per well and incubated at 37 °C for 1 h. Then, the HRP-labeled goat anti-mouse IgG antibody (BOSTER Biological TECHNOLOGY, Pleasanton, CA, USA) was diluted at a ratio of 1:6000 with 1 × PBS, and 100 μL was added to each well and incubated at 37 °C for 1 h, followed by thoroughly washing. A total of 100 μL tetra-methylbenzidine (TMB, Beyotime Biotechnology, Shanghai, China) chromogenic solution was added to each well and incubated at 37 °C for 15 min, and then 50 μL of chromogenic stop solution was added. The ELISA plate wells were measured using an enzyme-labeled instrument (Exl800, BioTek, Winooski, Vermont, United States) with OD_450_ value. When the OD_450_ value of the sample to be tested/the OD_450_ value of the negative sample (P/N) was ≥2.1, it was determined as positive.

### 2.6. Western Blot Analysis

The specificity of the monoclonal antibody was detected by Western blotting in reaction with the S1 antigen of the IBV M41 strain. CEK cells cultured in 12-well plates were infected with IBV at a multiplicity of infection (MOI) of 1 and incubated at 37 °C for 1 h, in which cells inoculated with phosphate-buffered saline (PBS) served as the negative control. At 36 h post-inoculation, cell proteins were collected using 200 μL of cell lysate (Solabio, Beijing, China). The protein samples (10 μL) of CEK cells were separated by 10% SDS-PAGE and transferred onto a polyvinylidene fluoride membrane. After blocking with 5% skimmed milk containing phosphate-buffered saline with tween (PBST) for 1 h, the membrane was incubated with the selected mAbs at 4 °C overnight. Then, the supernatant of the hybridoma cells was diluted at a ratio of 1:10 and used as the primary antibody. Subsequently, the membrane was incubated with horseradish peroxidase-labeled goat anti-mouse IgG antibody diluted in 5% skimmed milk at 37 °C for 1 h. Finally, the membrane was subjected to chemiluminescence detection using Clarity™ Western ECL Substrate (Bio-Rad, Hercules, CA, USA). The membrane was then immediately imaged using a Tanon 5200 Multi Imaging System (Tanon, Shanghai, China).

### 2.7. Indirect Immunofluorescence Assay (IFA)

CEK cells were subjected to indirect immunofluorescence staining using the supernatant of hybridoma cells, with or without MOI = 1 IBV (M41 strain) infection. After 48 h, CEK cells were fixed with 4% paraformaldehyde for 10 min and subsequently treated with 0.1% Triton-X for 10 min. After washing, the cells were co-incubated with the hybridoma supernatant at 4 °C for 12 h. Subsequently, cells were incubated with CoraLite594-conjugated goat anti-mouse IgG (H+L) fluorescent antibody (SA00013-3, Proteintech) at 37 °C for 45 min and then stained with DAPI for 5 min. Finally, these cells were observed under a fluorescence microscope (Axiovert A1, Carl Zeiss AG, Oberkochen, Germany) to analyze the interaction between the virus and the screened monoclonal antibodies.

### 2.8. Overlapping Truncated S1 Gene Design and B-Cell Epitope Identification

To identify the B-cell epitopes in the S1 protein targeting these monoclonal antibodies, the fragment of the S1 gene was first divided into three segments: 38–190 AA (S1-38), 114–190 AA (S1-190) and 114–257 AA (S1-114). S1-138 and S1-190 were amplified via PCR using primers containing *Kpn* I and *Xba* I restriction sites, while S1-114 was amplified using primers containing *Kpn* I and *Hind* III restriction sites. Subsequently, three S1 segments were cloned into the pCold I vector. Once the recombinant protein was expressed, Western blotting was performed utilizing both the screened mAb and His-tagged antibody. For further localization of antigenic epitopes, overlapping sequences of the S1 protein-specifically 107–147 AA (S1-107) and 128–193 AA (S1-128) were cloned using primers containing *BamH* I and *Hind* III restriction sites, and then ligated into the prokaryotic expression vector pET-32a(+). The resulting recombinant plasmids were transformed into *E. coli* BL21 (DE3) for expression.

Ultimately, three polypeptides were synthesized at Sangon Biotech Co., Ltd. (Shanghai, China), coupled to a BSA carrier, and their corresponding antigenic epitopes were confirmed through Western blotting. The primers used for constructing these truncated S1 genes are listed in Table 1.

### 2.9. Bioinformatics Analysis

MEGA11 software was used to perform the homology analysis of the specificity of the obtained epitopes with 2A10, 4E9, and 5E12 mAbs. Additionally, utilizing Pymol 2.6, we modeled the IBV M41 S1 protein and predicted the distribution of identified antigenic epitopes within the S1 protein, while also analyzing the spatial characteristics and biological functions of these mapped epitopes.

## 3. Results

### 3.1. Cloning, Expression and Purification of Recombinant S1 Protein

**Figure 1:**
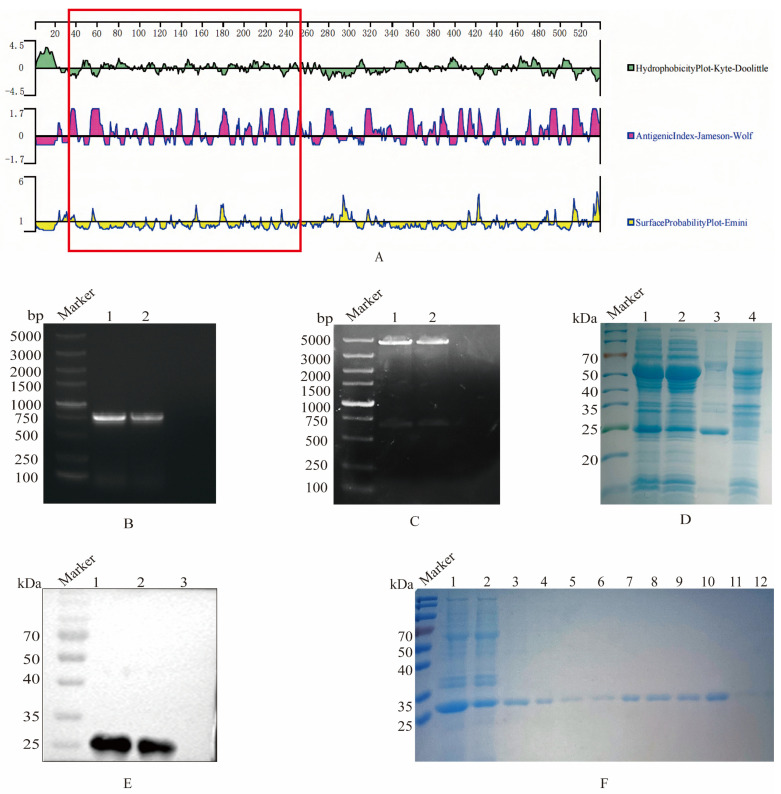
Selection, expression, and purification of the truncated S1 protein. (**A**) Structural characteristics of the S1 protein. The software Protean was employed to analyze the hydrophilicity, antigenic index, and surface probability of the S1 protein, with the region highlighted in the red box representing the selected fragment (38–257 AA). (**B**) PCR amplification of the truncated S1 gene. Lane 1 and 2, agarose electrophoresis of S1 gene amplification. (**C**) Identification of recombinant plasmid pCold I-S1 by double enzyme digestion. Lane 1 and 2, electrophoresis of pCold I-S1 after enzyme digestion. (**D**) S1 protein expression. The recombinant plasmid pColdI-S1 was transformed into BL21, and induced by IPTG, which was analyzed on SDS-PAGE. Lane 1, pCold I-S1 recombinant strain; Lane 2, supernatant after sonication of pCold I-S1 recombinant strain; Lane 3, precipitated after sonication of pCold I-S1 recombinant strain; Lane 4, pCold I recombinant strain control. (**E**) Specific reaction of recombinant S1 protein reacted with positive serum. Lane 1 and 2 pCold I-S1 (27.1 kDa); Lane 3, pCold I vector expression control. (**F**) Purification of recombinant S1 protein. Lane 1, pCold I-S1 recombinant strain; Lane 2, effluent after column hanging; Lane 3-12, effluent after elution with different imidazole concentrations. The imidazole concentrations were 50, 80, 100, 100, 120, 120, 150, 150, 150 and 250 mM.

The structural features of the S1 protein were analyzed with Protean 11.1 software, and the results revealed that the 38–257 AA region of the S1 protein exhibited strong hydrophobicity, antigenicity, and surface probability (Figure 1A). These characteristics indicated that this region might have good antigenic potential. Consequently, this segment was selected as the target S1 gene for the construction of recombinant plasmids.

Firstly, the truncated S1 gene was amplified by PCR, and the nucleic acid electrophoresis verified that the PCR production was in accordance with the expected size of 660 bp (Figure 1B). The results of the recombinant plasmid pCold I-S1 through double enzyme digestion confirmed that the S1 gene fragment was successfully ligated to the pCold I vector (Figure 1C). The SDS-PAGE results indicated that the recombinant pCold I-S1 protein was approximately 27 kDa, and the expression quantity was higher in the inclusion bodies (Figure 1D). Furthermore, the His tag antibody was employed as the primary antibody for Western blot validation, and a specific band at 27 kDa was observed, verifying the expression of the recombinant S1 protein (Figure 1E). Ultimately, after denaturation, the recombinant S protein was purified via nickel column affinity chromatography. At an imidazole concentration ranging from 100 to 150 mM, the recombinant S1 protein was effectively purified and a single target band was formed (Figure 1F). The S1 protein concentration was determined by the BCA assay to be 1.5 mg/mL.

### 3.2. Screening and Characterization of Monoclonal Antibodies

**Figure 2:**
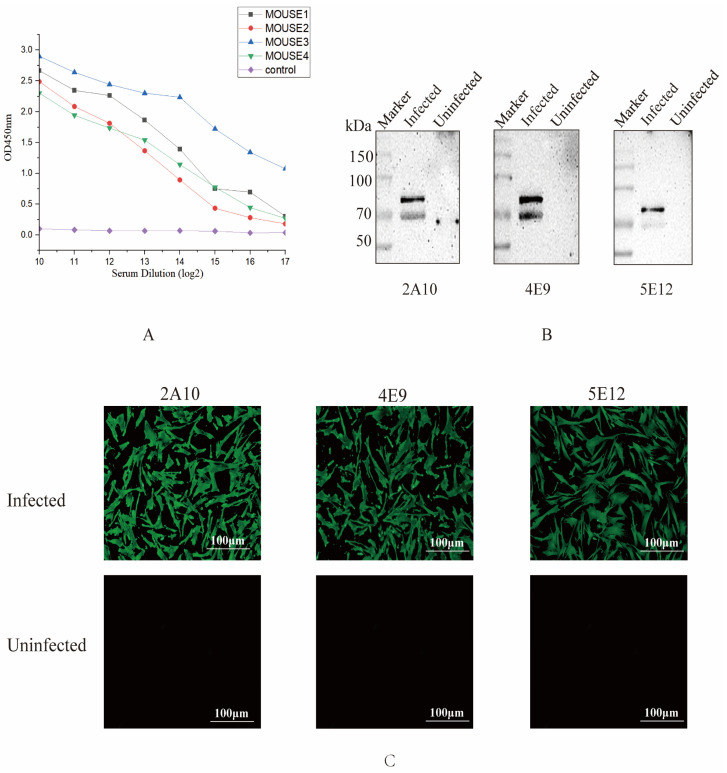
Screening and identification of monoclonal antibodies. (**A**) Antibody levels of the immunized mice. One week after the third immunization, the potency of antibodies from all mice was measured by indirect ELISA, and the results are presented as the absorbance value at OD_450_. (**B**) Recognition between mAbs 2A10, 4E9, and 5E12 and S1 protein. CEK cells were infected with IBV, and the protein samples were collected at 36 h to detect recognition between S1 protein and mAbs by Western blotting. (**C**) Reactivity of three mAbs detected by IFA. CEK cells were infected with IBV and subsequently incubated with selected mAbs to evaluate the reactivity of these mAbs against the S1 protein of IBV.

The sera of all immunized mice were collected, and the serum antibody levels were determined by indirect ELISA (Figure 2A). Among four immunized mice, the antibody level of mouse No. 3 was the highest. Subsequently, 200 μg of recombinant protein was used as an enhancer for booster immunization. After cell fusion and three rounds of subcloning, three hybridoma cell clones were identified by indirect ELISA, which secreted specific antibodies against the truncated S1 protein and were named mAb 2A10, 4E9, and 5E12. Then, the subtypes of monoclonal antibodies were identified, showing that the light chains of three antibodies (2A10, 4E9, and 5E12) were κ, while all the heavy chains were IgG1 (Table 4).

To determine whether the screened monoclonal antibodies specifically recognized the native conformation of the S1 protein, the cell supernatants obtained from the screened monoclonal cell lines were used to identify the protein samples of IBV M41-infected cells by Western blot. All three monoclonal cell supernatants could specifically recognize the native S1 protein (Figure 2B). Additionally, the results of the IFA experiment confirmed that monoclonal antibodies 2A10, 4E9, and 5E12 were capable of binding to virus-infected cells (Figure 2C), suggesting that these monoclonal antibodies might have the ability to recognize the S1 protein.

### 3.3. Identification of B-Cell Epitopes of Monoclonal Antibodies

**Figure 3:**
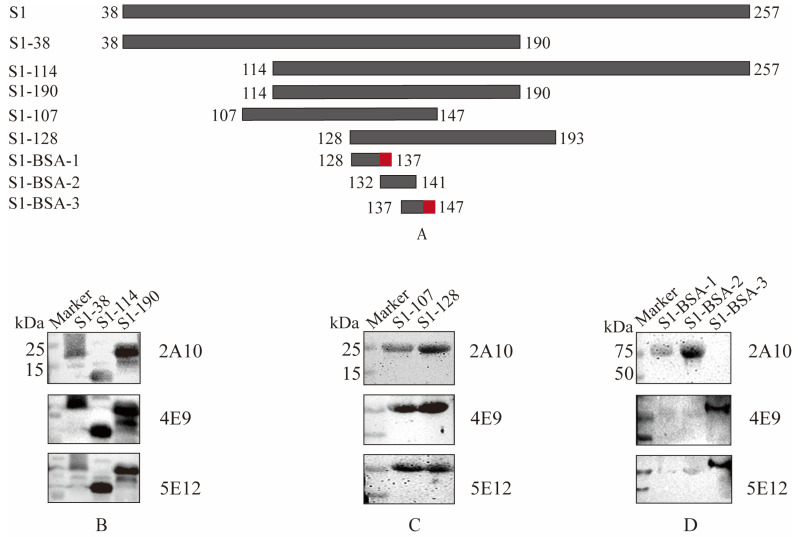
Identification of B cell epitopes of mAbs specific to S1 protein. (**A**) Schematic diagram of S1 epitope identification. The red-labeled regions represent the final identified antigenic epitope fragments. (**B**) Differentiation among truncated S1-38, S1-114, and S1-190 with three screened mAbs. (**C**) Differentiation between truncated S1-107 and S1-128 with three screened mAbs. (**D**) Identification of the coupled peptides with mAbs following Western blot analysis. The reactivities of mAbs with different truncated S1 proteins are shown in Table 5.

To ascertain the epitope in the S1 protein recognized by the screened monoclonal antibodies, four truncated proteins and three peptides based on the S1 protein sequence were constructed and synthesized in accordance with the immunogenic epitope database (http://tools.iedb.org/bcell/ (accessed on 25 February 2025)) (Figure 3A). Firstly, S1 (38–257 AA) was divided into three truncated genes, S1-38 (38–190 AA), S1-114 (114–257 AA), and S1-190 (114–190 AA). After PCR amplification, these three truncated genes were cloned into pCold I and expressed. The results of Western blotting showed that recombinant S1-38, S1-114, and S1-190 proteins were recognized by mAb 2A10, 4E9, and 5E12 (Figure 3B). To further confirm the epitopes recognized by the screened monoclonal antibodies, two truncated fragments S1-107 and S1-128 were designed and constructed. The genes of the two truncated fragments were amplified and cloned into pET-32a(+) for prokaryotic expression. The results of Western blotting showed that both recombinant S1-107 protein and S1-128 protein were recognized by monoclonal antibodies 2A10, 4E9, and 5E12 (Figure 3C).

To determine the minimal epitope of the S1 protein, three peptides ranging from 128 to 147 AA, ^128^QHSIRVSAMK^137^, ^132^RVSAMKNGQL^141^, and ^137^KNGQLFYNLTV^147^, with overlapping amino acids, were synthesized and conjugated to BSA. The results of Western blotting showed that peptides ^128^QHSIRVSAMK^137^and ^132^RVSAMKNGQL^142^ were recognized by 2A10 mAb, and peptide ^137^KNGQLFYNLTV^147^ was recognized by 4E9 and 5E12 mAbs (Figure 3D). Generally, B-cell epitopes have at least five amino acids. For example, 2A10 recognizes polypeptides S1-BSA-1 and S1-BSA-2, and the overlapping amino acids between S1-BSA-1 and S1-BSA-2 are 6 AA, as shown in Table 5. Therefore, 132–137 AA is the possible epitope recognized by 2A10. 4E9 and 5E12 can recognize S1-BSA-2 but not S1-BSA-3, and their overlapping fragment is 5 AA (137–142 AA), indicating that the possible recognition region of 4E9 and 5E12 is at 142–147 AA. Therefore, these results suggested that in the S1 protein of IBV M41, 2A10 mAb might recognize ^132^RVSAMK^137^, while 4E9 and 5E12 may recognize ^142^FYNLTV^147^.

### 3.4. The Identified Epitopes Are Highly Conserved in IBV Strains

**Figure 4:**
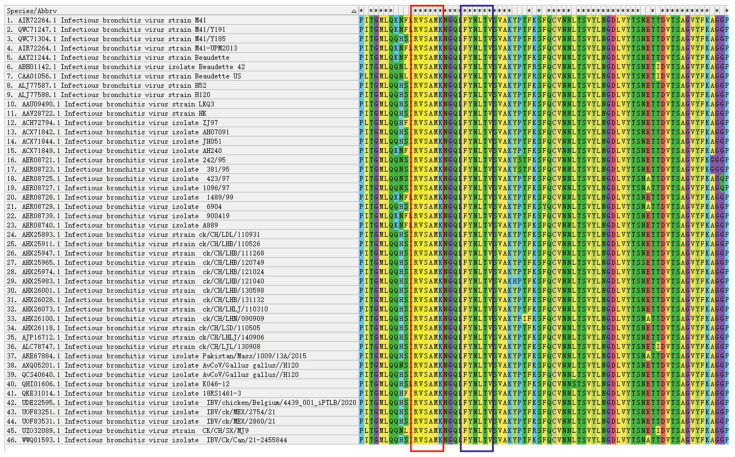
Comparison of antigenic epitopes in S1 protein. Homology analysis of the selected sequences was performed in 56 strains by the software MEGA-11. The antigenic epitope ^132^RVSAMK^137^ is marked in the red box and the antigenic epitope ^142^FYNLTV^147^ is marked in the blue box.

A comparative homology analysis was conducted among various subtypes to determine the conserved properties of the identified antigenic epitopes (Figure 4). The epitopes ^132^RVSAMK^137^ and ^142^FYNLTV^147^ were conserved in the S1 protein of 46 IBV strains.

### 3.5. Spatial Location Prediction of Epitope Binding

**Figure 5:**
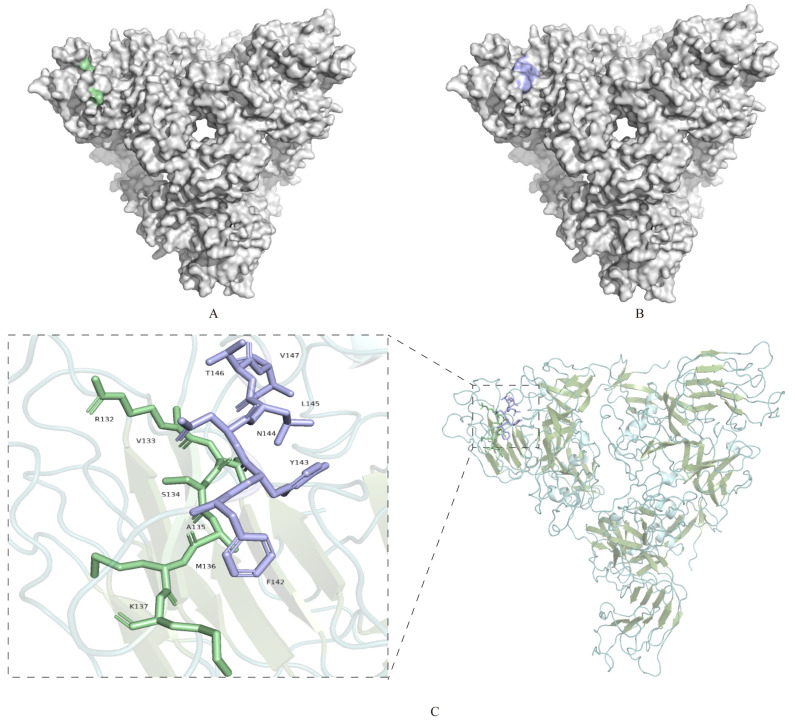
Localization of the identified S1 protein-specific epitopes. (**A**) Epitopes identified ^132^RVSAMK^137^ by 2A10 mAb are marked in pale green on the surface of the S1 protein. (**B**) Epitopes ^142^FYNLTV^147^ identified by 4E9 and 5E12 mAbs are marked in light blue on the surface of the S1 protein. (**C**) Overall structure of S1-NTD, displayed as a cartoon. α-helices are shown in pale cyan, β-sheets in smudge, and loop regions in light teal. The epitope ^132^RVSAMK^137^ located within a β-sheet region, is highlighted in pale green. The epitope ^142^FYNLTV^147^, located within a loop region, is highlighted in light blue. Boxed regions indicate the areas magnified in the insets, highlighting the secondary structures where the epitopes are located. Insets: Close-up views of the boxed regions, showing the detailed structures of the epitopes ^132^RVSAMK^137^ (β-sheet) and ^142^FYNLTV^147^ (loop).

The spatial locations of the two identified epitopes, ^132^RVSAMK^137^ and ^142^FYNLTV^147^, as predicted by the modeling results and the antigen epitopes, were analyzed using Pymol 2.6 software and are presented in Figure 5. The epitope ^132^RVSAMK^137^ was situated on the protein surface (Figure 5A), and ^142^FYNLTV^147^ was also located on the protein surface (Figure 5B). These two epitopes were positioned on the surface of the S1 protein at distinct locations, whose dynamic process of spatial structure of these sites was predicted (Video S1). The epitope ^132^RVSAMK^137^ was identified within a β-sheet region, while the epitope ^142^FYNLTV^147^ was located in a loop structure. These discoveries might have implications for the development of diagnostic assays and therapeutic approaches for IBV infection.

## 4. Discussion

IBV is currently ubiquitous worldwide and has been prevalent. The S1 gene in IBV M41 is one of the genes that undergo frequent alterations, thereby posing greater challenges for vaccination-induced protection [24]. The S1 protein, as a subunit of the spike protein, plays an irreplaceable role in viral invasion of the host and immune evasion. Numerous critical amino acid sites on the S1 protein can directly influence the recognition of the virus with the receptor on the surface of host cells [25]. Research indicates that replacing the S gene in the Beaudette background with the IBV M41 strain restricts the tropism of the virus for primary chicken cells [26]. This information suggests that further exploration is necessary to screen the functions and performances of the determinant sites in the S1 protein.

Identifying the key determinant sites of the S1 protein not only contributes to the improvement of disease detection techniques but also helps to reveal the functional targets of the S1 protein during the virus’s invasion of cells. In this study, through DNASTAR software analysis, the S1 region at 38–257 AA has a strong hydrophilic region with high surface probabilities and strong antigenicity, which is a relatively conserved domain. Then, the truncated fragment of the IBV M41 S1 protein was cloned for prokaryotic expression. Eukaryotic expression is a commonly used genetic engineering technique in the preparation of monoclonal antibodies and the identification of antigenic epitopes [27]. Our data showed that the recombinant protein was expressed at a high level in the form of inclusion bodies. After fusion with mouse cells immunized with the recombinant S1 protein, three hybridoma cell clones—2A10, 4E9, and 5E12—that secrete specific antibodies targeting the S1 protein were screened through indirect ELISA, Western blotting, and indirect immunofluorescence. The determination of antigenic epitopes is conducive to the creation of epitope vaccines and the understanding of the molecular mechanisms of the virus [28]. Analyzing the antigenic epitopes of IBV prevalent strains is beneficial for a better understanding of the antigenic variations of IBV during its genetic evolution, thereby facilitating the control and elimination of IBV [29]. In early studies, the identification of antigenic epitopes primarily relied on sequence analysis. Through a systematic comparison of the amino acid sequences and secondary structures of the S1 protein from 24 IBV strains, Wang et al. successfully identified a highly conserved epitope located at the N-terminus, with the sequence ^240^QYNYGYNFPSEDGFWPTN^255^ [30].In this paper, two B cell epitopes precisely localized using truncation and peptide scanning procedures, namely ^132^RVSAMK^137^ and ^142^FYNLTV^147^, are conserved among various subtypes of IBV strains and are located on the protein surface, and have not been previously reported in the literature. Most epitope mapping studies on the IBV S1 protein have primarily targeted the receptor-binding domain within the S1-CTD [31,32]. Bande et al. predicted, through the analysis of relevant bioinformatics software, that the 80–89 AA position of the S1 gene contains potential B cell epitopes [33]. Ignjatovic et al. demonstrated that the two regions 294–316 (Sp4) and 532–537 (Sp6) in the S1 protein, which can be recognized by IBV immune sera, contain B cell epitopes that can induce a favorable immune response in the body [34]. Zou Nianli et al. identified the linear B cell epitopes ^87^PPQGMAW^93^ and ^416^IQTRTEP^422^ through phage display peptide library technology [35]. Due to the high sequence diversity of the receptor-binding domain (RBD), monoclonal antibodies (mAbs) targeting this region are often limited by restricted cross-reactivity, and the specific secondary structural locations of these epitopes remain poorly defined. In this study, the monoclonal antibodies were developed against the S1-NTD, a region that has not been extensively explored. Through predictive structural analysis, the epitope ^132^RVSAMK^137^ was identified to reside within a β-sheet region, further supporting its high conservation. This structural feature not only enhances the stability of antibody binding but may also improve the neutralizing activity of the antibodies, contrasting sharply with the α-helical epitopes commonly found in the S1-CTD, which are prone to viral escape mutations. On the other hand, the ^142^FYNLTV^147^ epitope is located in a loop region, whose flexibility provides a unique advantage for antibody binding. Amino acid residues in this region typically exhibit high dynamics, enabling them to adapt to conformational changes during antibody binding. Loop regions often mediate protein-protein interactions and conformational transitions, suggesting that targeting this epitope could disrupt viral entry by locking the S1-NTD in an inactive state or interfering with the membrane fusion process [36].

By identifying and utilizing these epitopes, it is possible to design and construct candidate multivalent epitope vaccines, or develop drugs that can activate or modulate immune responses. Moreover, targeting the stable β-sheet and the dynamic loop region provides a dual mechanism of action. The conserved nature of the β-sheet epitope makes it an ideal target for developing broad-spectrum diagnostic tools, while the surface exposure and flexibility of the loop epitope can serve as a sensitive biomarker for early infection detection. These characteristics offer unique structural advantages for antibody binding and hold significant potential for the development of antibody-based drugs. These findings provide targets for researching and establishing rapid detection methods for avian infectious bronchitis and also offer new biological materials for exploring the functions of the S1 protein. They open up broad possibilities for future research and applications and are expected to play an important role in the prevention and control of IBV.

## Figures and Tables

**Table 1:** Primers of the truncated S1 gene for PCR cloning.

Gene Name	Primer Name	Primer Sequence (5′–3′)	Primer Size	Product Size (bp)
S1	S1-F	TCGGTACCGATGGTTGGCATTTACATGG	28	660
	S1-R	ACAAGCTTGACAATAAACTTCTGCTTAAC	29	
S1-38	S1-38-F	TCGGTACCGATGGTTGGCATTTACATGG	28	459
	S1-38-R	CTATCTAGAAAAATAAACACCTGCAGATGT	30	
S1-114	S1-114-F	CTGGTACCCATGTTGGGTGTCCTATAAC	28	423
	S1-114-R	ACAAGCTTGACAATAAACTTCTGCTTAAC	29	
S1-190	S1-190-F	CTGGTACCCATGTTGGGTGTCCTATAAC	28	231
	S1-190-R	CTATCTAGAAAAATAAACACCTGCAGATGT	30	
S1-107	S1-107-F	TCGGATCCACTACAGTGTTTGTTACACATT	30	198
	S1-107-R	GCAAGCTTATCACCATTTAAATATACGGATGT	32	
S1-128	S1-128-F	TCGGATCCCAACAGCATTCTATACGTGTT	29	198
	S1-128-R	GCAAGCTTAGCTTTAAAATAAACACCTGC	29	

Note: Horizontal characters were restriction endonuclease sites, as follows: GGTACC, *Kpn* I; GGATCC, *BamH* I; AAGCTT, *Hind* III; TCTAGA, *Xba* I.

**Table 2:** PCR system.

Component	Volume (µL)	Concentration
PrimeSTAR HS Premix	25	2×
Primer-F	1	10 µM
Primer-R	1	10 µM
cDNA	1	100 ng/µL
ddH_2_O	Add to 50	

**Table 3:** PCR reaction program.

Reaction Basic Steps	Temperature (°C)	Time	Cycles
Initial denaturation	98	2 min	1
Denaturation	98	10 s	35
Annealing	56	5 s	35
Extension	72	1 min	35
Final Extension	72	5 min	1

**Table 4:** Monoclonal antibody isotype identification and antibody potency.

mAbs	Antibody Potency of Supernatant	Heavy Chain	Light Chain
2A10	1:6400	IgG1	Kappa
4E9	1:12,800	IgG1	Kappa
5E12	1:1600	IgG1	Kappa

**Table 5:** The reactivities of three monoclonal antibodies with the truncated S1 protein.

Vector Truncation	mAbs		
	2A10	4E9	5E12
S1-38	+	+	+
S1-114	+	+	+
S1-190	+	+	+
S1-107	+	+	+
S1-128	+	+	+
Peptide S1-128	+	-	-
Peptide S1-132	+	-	-
Peptide S1-137	-	+	+

Note: “+” is reactivity; “-” is no reactivity.

## Data Availability

The data presented in this study are available upon request from the corresponding author.

## Supplementary Materials

The following supporting information can be downloaded at: https://www.mdpi.com/article/10.3390/v17040464/s1, Video S1: Visualization of Spatial Distribution of Antigenic Epitopes in the 3D Model of the S Protein.
