# Supplementary figures and images for: Identification of B-Cell Epitopes Located on the Surface of the S1 Protein of Infectious Bronchitis Virus M41 Strains
Source: Viruses. 2025 Mar 24;17(4):464. doi: 10.3390/v17040464 (PMC12031124; doi:10.3390/v17040464)

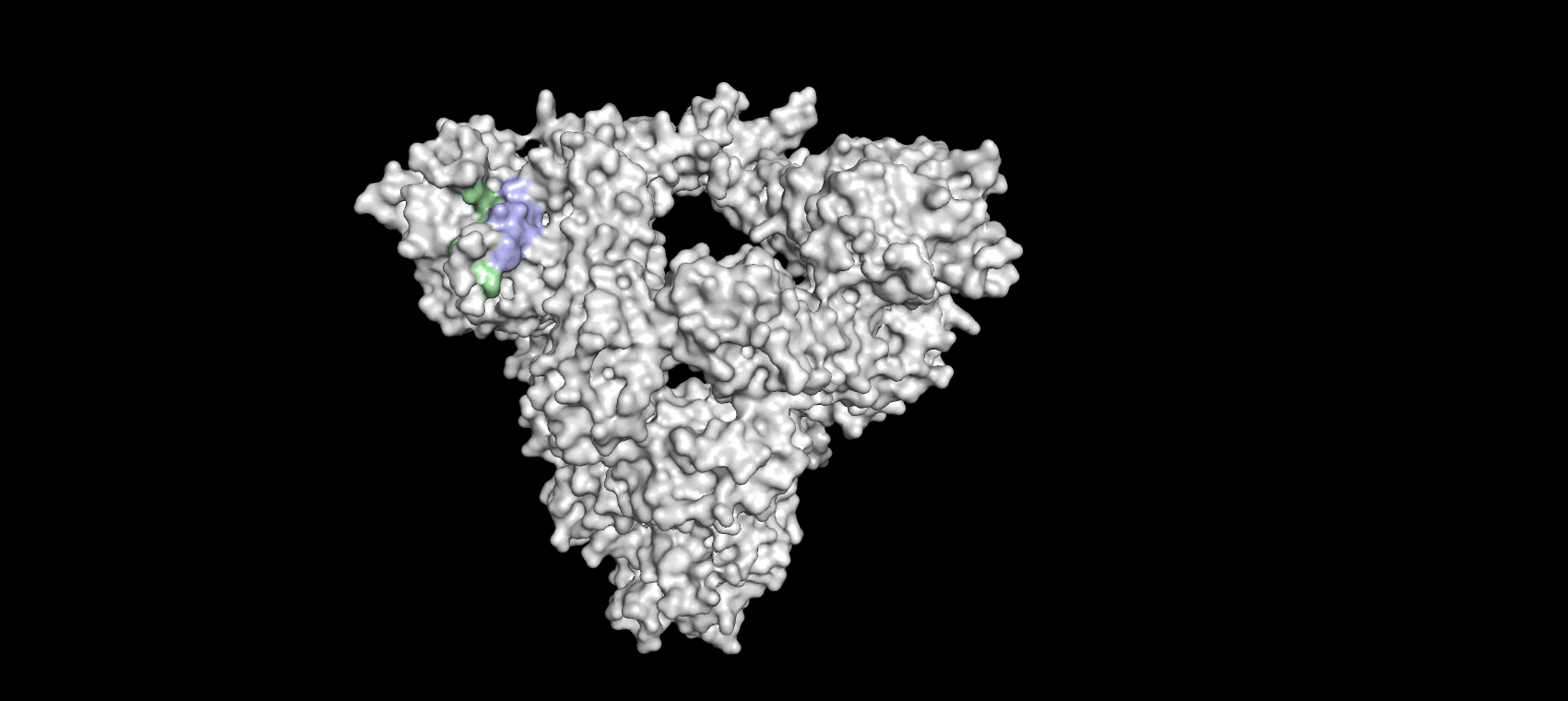

Supplement: Supplementary file 1 [file viruses-17-00464-s001.zip › Additional Material 1.gif]
